# Environmental and biological factors that influence feeding behavior of Holstein calves in automated milk feeding systems

**DOI:** 10.3168/jdsc.2023-0374

**Published:** 2023-07-13

**Authors:** Maria E. Montes, Jarrod Doucette, Luiz F. Brito, Jacquelyn P. Boerman

**Affiliations:** a Department of Animal Sciences, Purdue University, West Lafayette, IN 47907; b Ag Data Services, Purdue University, West Lafayette, IN 47907

## Abstract

Automated milk feeders (AMF) used for dairy calves continuously provide individual feeding behavior measurements. The objective of this retrospective cohort study was to evaluate the association between temperature-humidity index (THI), birth weight, and dam parity characteristics on feeding behavior (i.e., milk consumption and drinking speed). Historical data sets generated from a single commercial dairy farm, where healthy (not treated for bovine respiratory disease, enteric disease, or injury) Holstein calves were fed up to 24 L/d of milk, were used for the analysis. A total of 5,312 female Holstein calves born between August 2015 and August 2021 (mean birth weight ± standard deviation: 40.7 ± 4.7 kg) on a commercial dairy farm were fed up to 24 L/d of nonsaleable milk for the first 32 d. For the analyses, feeding behavior data from the AMF system were combined with demographic data from the farm management software, and weather records from the closest public weather station (7 km away). Linear mixed models used to analyze daily milk consumption and drinking speed included THI, birth weight, dam parity, and feeding day as fixed effects, and feeder and calf within feeder as random effects. These models explained 57% of the total variation in milk consumption and 48% of the variation in drinking speed. Calves born from primiparous cows had the lowest milk consumption and the greatest drinking speed in comparison to calves born from multiparous cows. Calves with heavier birth weights had higher milk consumption and faster drinking speed than lighter calves. Drinking speed was negatively associated with THI. Including data derived from individual calves and their environmental conditions in data sets exploring feeding behavior from AMF would control for variation and improve the predictive models for performance assessment.

## Highlights

• Lighter calves had slower drinking speed and lower milk consumption.
• Calves from multiparous dams drank milk at a slower speed.
• Drinking speed and THI were negatively associated.

## Graphical Abstract

**Figure:**
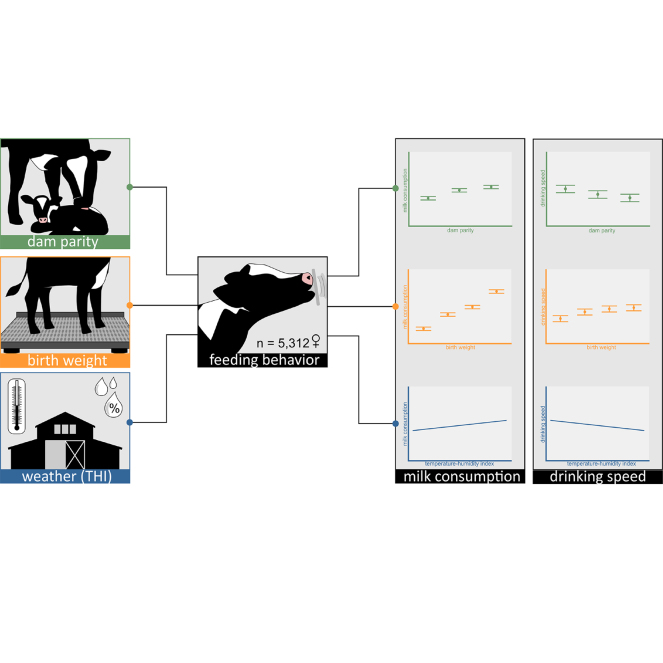
**Summary:** This study integrated and analyzed historic data sets from a commercial dairy farm to evaluate the association between the temperature-humidity index (THI), birth weight, and dam parity with the feeding behavior of female Holstein calves in automated milk feeder systems. The records of the first 32 feeding days from 5,312 calves, fed up to 24 L/day of milk, were analyzed using mixed models. The results showed that calves born from primiparous cows had the lowest milk consumption and the greatest drinking speed compared with those born from multiparous cows. Additionally, calves with heavier birth weights had higher milk consumption and faster drinking speed than lighter calves. Milk consumption and drinking speed were found to be positively and negatively associated with THI, respectively. These findings suggest that incorporating dam and weather data can control for variation, thereby improving predictive models for performance assessment of calves in automated milk feeder systems.

Although automated milk feeders (**AMF**) facilitate producers to deliver more milk to calves in multiple meals, raising healthy replacements still depends on cleanliness, adequate ventilation (Jorgensen et al., 2017), group management (Svensson and Liberg, 2006; Pedersen et al., 2009), and timely disease detection (McGuirk, 2008). Automated milk feeders collect longitudinal data on milk consumption, drinking speed, and the number of visits (rewarded and unrewarded) to the milk feeding station for individual calves. Calves raised in groups with AMF benefit from interacting with other calves (Costa et al., 2014). However, the rapid identification of sick animals remains a challenge in group settings (McGuirk, 2008). Changes in feeding behavior in preweaning dairy calves have been shown to indicate the onset of bovine respiratory disease (**BRD**; Knauer et al., 2017; Cantor and Costa, 2022), and enteric disease (Knauer et al., 2017; Conboy et al., 2022). However, disease detection should not rely solely on feeding behavior because it is not sensitive or specific (Knauer et al., 2018; Cramer and Ollivett, 2020). Beyond disease, environmental conditions (Dado-Senn et al., 2022), milk allowance, management, and social context can influence behavior of calves (Miller-Cushon and DeVries, 2015).

Reduced drinking speed and milk consumption are not specific to sickness; ambient temperature also influences energy needs (NASEM, 2021) and affects respiration rates of calves (Ferraro et al., 2021). Heat-stressed calves consume less milk (Dado-Senn et al., 2022) and have higher respiration rates (Dado-Senn et al., 2020) than calves cooled with fans. Moreover, due to their physio-anatomical traits and genetic background, individuals respond differently to external stressors (NRC, 1971a). Because changes in feeding behavior are not specific to sickness, accounting for the effects of environmental and biological factors could improve predictive models (Cantor et al., 2022b).

Integrating data from multiple sources in commercial dairies provides the context needed to derive meaningful information for supporting decisions (Cabrera et al., 2020). The objective of this study was to use historical data sets generated on a commercial farm to evaluate the association between temperature-humidity index (**THI**), birth weight, and dam parity on feeding behavior (i.e., milk consumption and drinking speed) when female Holstein calves are fed up to 24 L/d of milk and are not being treated for BRD, enteric disease, or injury. We hypothesized that increasing the parity of the dam and birth weight of the calf would increase milk consumption and drinking speed, and THI would be inversely related to milk consumption and drinking speed.

We conducted a retrospective cohort study with data from a commercial dairy farm located in Plymouth, Indiana. Animal data generated from January 2015 and August 2021 were collected from the AMF and the herd management software. Statistical power analyses were not used to define the study sample size as all the data available that passed the filtering criteria were kept for subsequent analyses. This study used a convenience sample of 5,312 female Holstein calves, with calf considered as the experimental unit. The study did not involve any procedures that altered the operation of the farm or affected calf behavior, and therefore, it was exempt from Institutional Animal Care and Use Committee approval.

The farm contained 4 calf barns, each with 4 pens and 2 AMF (DeLaval CF1000). Each AMF supplied milk to 2 pens through 4 feeding stations, 2 feeding stations per pen. Pens had a surface of approximately 223 m^2^ (minimum resting surface of 3.5 m^2^ per calf) and were ventilated with 2 positive-pressure tubes year-round. Curtains in the barns were manually adjusted according to the outside temperature such that at 15.6°C, all the curtains were open. At temperatures below 15.6°C, curtains and side doors closed gradually until the temperature reached −6.7°C, when all curtains were closed. The farm grouped calves using an all-in-all-out system reaching a maximum of 53 ± 10 (mean ± SD) calves in one pen. Groups consisted of female and male calves that were purebred Holstein or dairy-beef cross. Before being enrolled in the AMF, calves received 2 feedings of colostrum. Soon after birth, they received 3.8 L of colostrum through an esophageal feeder and 1.9 L 6 h later. Calves were then moved to the AMF where calf managers trained them during the first few days and ensured that they consumed milk via the AMF. Calf managers measured the calves' weights using a scale and recorded them in the herd management software. Weights recorded on the day of birth or the day afterward were considered the birth weight for this study.

The AMF delivered pasteurized milk enhanced with 20 g/L of milk powder (30% protein and 5% fat). During the first 32 feeding days calves had access up to 24 L of milk daily, dosed in portions with 2-h intervals that varied depending on the feeding day. On feeding d 0–10, calves could consume up to 2 L every 2 h; on feeding d 11–20, 2.5 L every 2 h; and on feeding d 21–32, 3 L every 2 h. Milk allowance gradually decreased after feeding d 32 until calves were fully weaned by feeding d 60. This study aimed to analyze feeding behavior when calves had access to near ad libitum milk allowance; therefore, only data from the first 32 feeding days were used. During the entire time, calves had ad libitum access to water and concentrate (customized blend: 18% CP and 3.5% crude fat). Concentrate was distributed in a bunk feeder located inside the pen and intake was not recorded.

The farm provided the AMF data from January 2015 to February 2019 (section 1) that were collected through the KalbManagerWIN software (version 3.5, Förster-Technik, Engen, Germany). Data were retrieved directly from the AMF cloud server (Förster-Technik) from August 2019 to August 2021 (section 2). DairyComp305 (Valley Ag Software) backups were used for records of birth date, birth weight, sex, breed, parity of the dam, and any medical treatments. Historical weather data of a nearby (7 km) weather station were available at Visual Crossing (Reston, VA) for Application Programming Interface download.

In this 6-yr dataset, 24,658 unique calves were identified by pairing ear-tag with registration number or Förster-Technik life number. The AMF data were integrated with the demographic data based on ear-tag, birth date, and registration number when available. After data integration, calves were removed from the dataset if they were missing at least 5 daily records (n = 8,943), were males or dairy-beef crosses (n = 1,619), were missing birth weight (n = 350), their birth weight was outside 4 SD from the mean (n = 13), had been assigned to a different milk feeding plan (n = 350), or had been treated for BRD, enteric disease, or injury at least once (n = 4,425). The final sample consisted of 5,312 healthy purebred female Holstein calves.

Duplicated records for calf and day were removed from the final data set (n = 284). Daily records were removed if the number of rewarded visits was zero (n = 797) or daily milk consumption was less than 1 L (n = 1,336) to avoid inaccurate feeder readings. With the same purpose, drinking speed was set as missing if the recorded value was zero (n = 204) or outside 4 SD from the mean of the corresponding feeding day (n = 595).

Temperature-humidity index was estimated following the US NRC equation (Equation 1; NRC, 1971b), with the values of relative humidity (RH, %) and average dry bulb temperature (T_db_,°F) at 2 m above surface level for each day (Visual Crossing Support, personal communication, August 8, 2022).

[1] THI = (T_db_) − [(0.55 − 0.55RH) × (T_db_ − 58)]

The variables dam parity and birth weight were considered as categorical in all the analyses. Within the sample, the parity of the dams ranged from 1 to 7. Only 7% of the calves were born from cows of fourth calving or higher. Therefore, the categories for dam parity used were 1, 2, and 3 or more. Birth weight was divided into categories according to quartiles with ranges 22–38, 38–41, 41–44, and 44–59 kg. Dam parity 1 and the lightest quartile for birth weight were considered the referent population. The purpose of doing quartile classification was to avoid assuming the shape of the relationships between birth weight and feeding behavior variables, but rather to identify if relationships existed between birth weight and milk feeding behavior.

All statistical analyses were conducted using the R software version 4.1.2 (R Core Team, 2021) in RStudio environment version 2022.7.1.554 (RStudio Team, 2022). The distribution of the feeding behavior variables was determined by plotting both daily and aggregated values. To ensure that all levels of birth weight and dam parity were represented across the time frame, plots from random subsamples at different fractions of time (i.e., year, season, section) were generated, assessed, and deemed appropriate for further analyses.

The analysis of daily milk consumption and drinking speed consisted of a linear regression mixed model fitted with the R package lmerTest (Kuznetsova et al., 2017). Both models included the random effects of calf nested in feeder, and the fixed effects of feeding day, birth weight, dam parity, and THI. Only those variables with *P* ≤ 0.01 remained in the final model. The least squares means for the categorical variables and the predicted values across the observed range of THI were estimated using the emmeans package (Lenth, 2022). Differences in milk consumption and drinking speed between dam parity and birth weight levels were determined based on Tukey-adjusted pairwise comparisons at *P* ≤ 0.01.

Summary statistics consisted of average daily milk consumption and average daily drinking speed over the 32-d period. Calves consumed on average 9.3 ± 1.4 L (mean ± SD) of milk daily and had an average drinking speed of 499 ± 86.3 mL/min (mean ± SD). Average daily milk consumption ranged from 4.2 L to 15 L. Average drinking speed ranged from 180 mL/min to 912 mL/min. Although the mean daily milk consumption and drinking speed increased from d 0 to d 32, milk consumption increased at a faster rate in the first 3 feeding days and drinking speed showed a negative slope between feeding d 3 and 5. The minimum THI in the data set was 0.3 and the maximum was 79.3, and the average was 52.0.

All the variables included in the model (i.e., feeding day, birth weight, dam parity, and THI) were associated (*P* < 0.01) with daily milk consumption (Table 1). These variables accounted for a combined 57% (conditional R^2^) of the variation. When first enrolled in the AMF system (feeding d 0), calves consumed 5.8 ± 0.05 L of milk and increased their consumption by 0.22 ± 0.01 L each feeding day up to 12.9 ± 0.05 L by 32 d on the feeding plan. Calves with heavier birth weights consumed more milk per day. Animals were separated into quartiles based on birth weight and each quartile consumed at least 0.30 L more than the lighter one (20–38 kg: 8.80 ± 0.05 L vs. 38–41 kg: 9.24 L vs. 41–44 kg: 9.54 L vs. 44–59 kg: 10.04 L; all *P* < 0.01; Figure 1a). Independent of birth weight, calves born from multiparous cows consumed more milk per day than calves born from primiparous cows (parity 1: 9.19 ± 0.05 L vs. parity 2: 9.48 L vs. parity 3+: 9.55 L; all *P* ≤ 0.01; Figure 1c). Even though THI was associated with daily milk consumption, the effect size was small (0.005 ± 0.01 L; Figure 2a). At the lowest THI (0.3) the predicted daily milk consumption was 9.1 ± 0.06 L, and at the highest THI (79.3) 9.5 ± 0.05 L.

**Table 1:** Estimated linear coefficients^1^ of biological and environmental factors on the feeding behavior of preweaned female Holstein calves (n = 5,312) in an automated milk feeding system

Variable	Milk consumption, L			Drinking speed, mL/min		
	Estimate	SE	*P-*value	Estimate	SE	*P-*value
Intercept	4.69	0.07	<0.01	392.28	8.62	<0.01
Temperature-humidity index	0.01	<0.01	<0.01	−0.40	0.04	<0.01
Feeding day	0.22	<0.01	<0.01	7.59	0.03	<0.01
Dam parity						
1		—	—		—	—
2	0.29	0.05	<0.01	−9.73	2.9	<0.01
3 and >3	0.36	0.05	<0.01	−18.34	3.12	<0.01
Birth weight,^2^ kg						
20–38		—	—		—	—
38–41	0.43	0.05	<0.01	17.58	3.39	<0.01
41–44	0.74	0.05	<0.01	24.70	3.39	<0.01
44–59	1.24	0.05	<0.01	26.30	3.42	<0.01

1 Results from the linear mixed model fitted with the R package lmerTest (Kuznetsova et al., 2017) considering the random effects of calf nested in feeder.

2 Weight categories correspond to quartiles.

**Figure 1:**
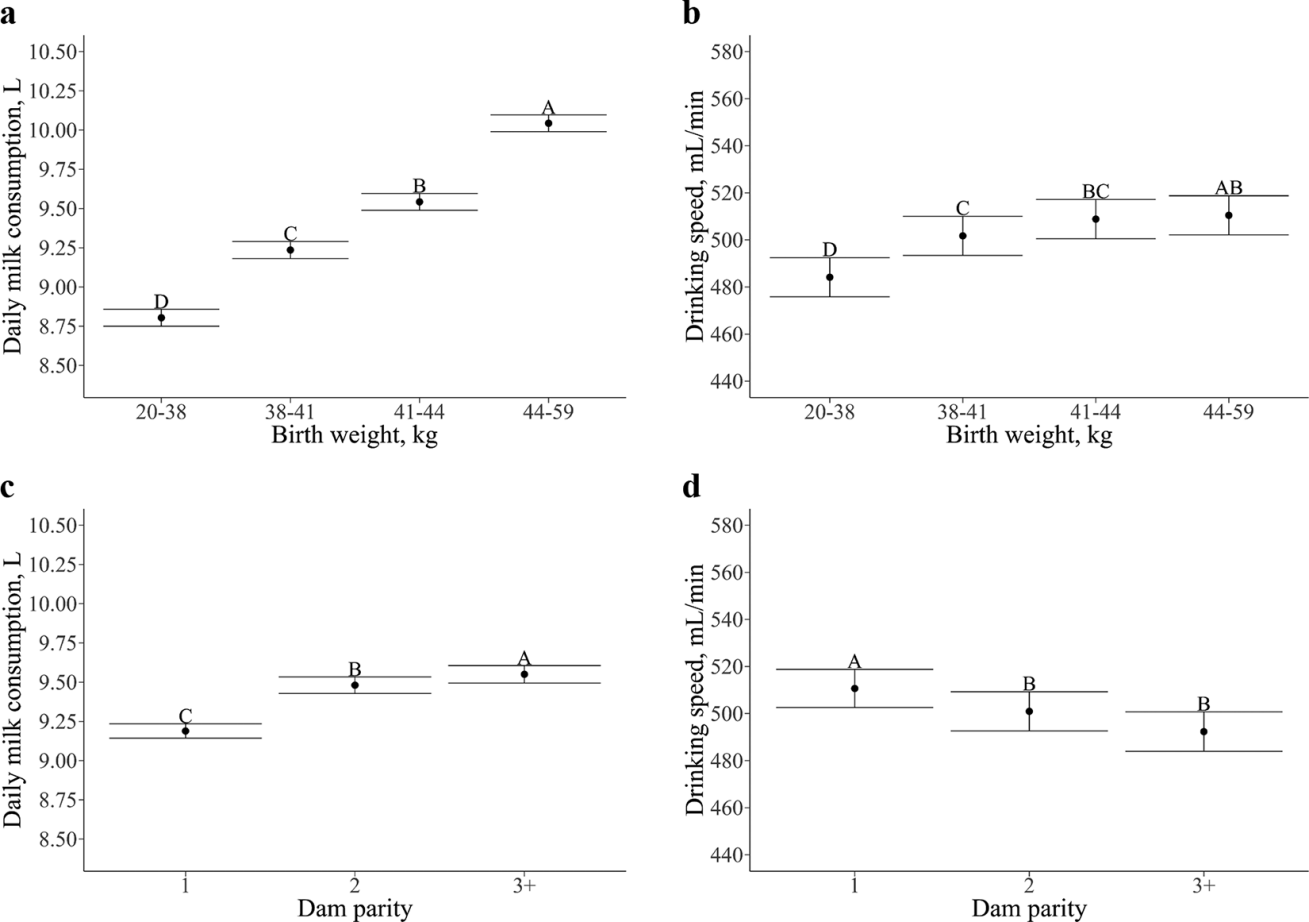
Estimated LSM and SE of (a) daily milk consumption by birth weight, (b) drinking speed by birth weight, (c) daily milk consumption by parity, and (d) drinking speed by parity on preweaning female Holstein calves (n = 5,312) in an automated milk feeding system. Letters indicate significance (*P* ≤ 0.01) in Tukey-adjusted pairwise comparisons. Birth weight categories correspond to quartiles. Parity categories correspond to first parous, second parous, and third parous or higher.

**Figure 2:**
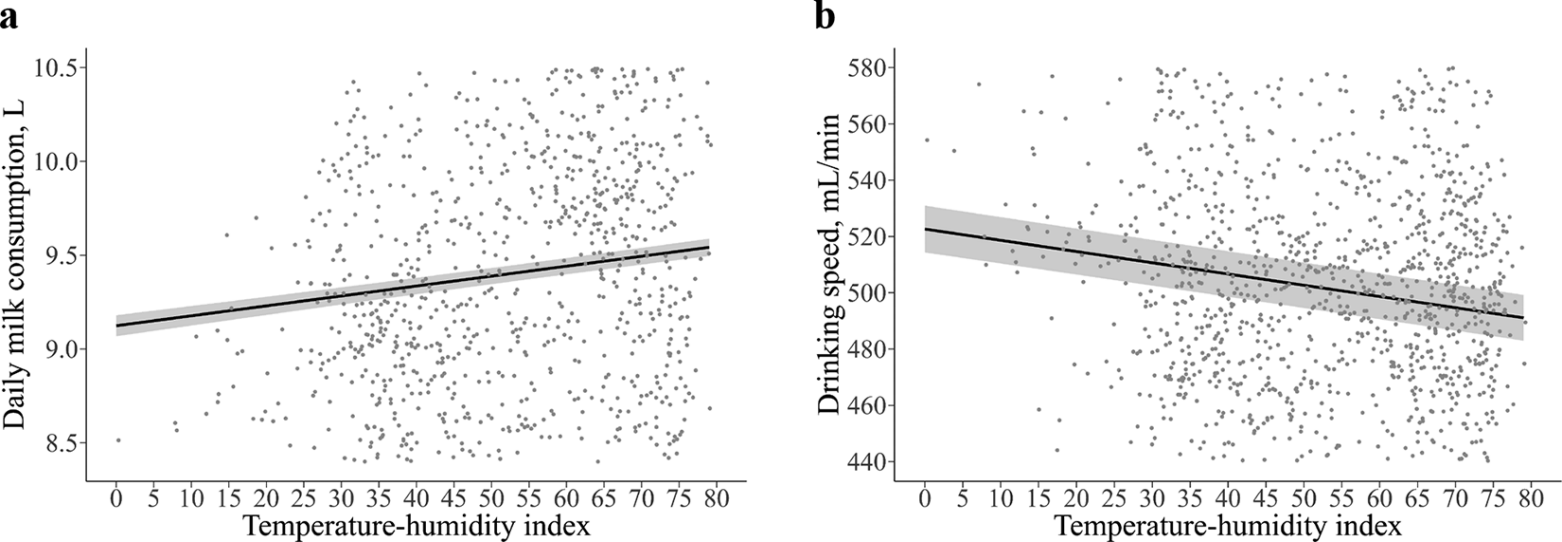
Effect of temperature-humidity index on (a) daily milk consumption (*y* = 9.12 + 0.005*x*, *P* < 0.01), and (b) drinking speed (*y* = 523 − 0.399*x*, *P* < 0.01), of preweaned female Holstein calves (n = 5,312) in an automated milk feeding system. The line represents the predicted values for the population, and the shaded area corresponds to the standard errors of the prediction. Dots are random subsets of 3,200 fitted values.

Similarly, all the observed variables (i.e., feeding day, birth weight, dam parity, and THI) were associated (*P* < 0.01) with daily drinking speed (Table 1). These variables accounted for a combined 48% (conditional R^2^) of the total variation. When first enrolled in the AMF system (feeding d 0), calves drank milk at 379 ± 8.07 mL/min and increased by 7.6 ± 0.03 mL/min each feeding day up to 622 ± 8.07 mL/min by 32 d on the feeding plan. Calves with birth weights in the lightest quartile (20–38 kg) had the slowest drinking speed (485 ± 8.33 mL/min; *P* < 0.01) compared with all other quartiles. Drinking speed was not different between the second (38–41 kg), third (41–44 kg), and fourth (44–59 kg) quartiles. However, calves with birth weights in the heaviest quartile drank milk at the fastest speed (511 ± 8.31 mL/min; Figure 1b). Calves born from multiparous cows drank slower than calves born from primiparous cows (parity 1: 511 ± 8.12 mL/min vs. parity 2: 501 mL/min L; *P* < 0.01; Figure 1d). However, there was no difference on drinking speed between calves born from second parity cows and those from third parity or higher. Even though THI was associated with drinking speed, the effect size was small (−0.4 ± 0.04 mL/min; Figure 2b). At the lowest THI (0.3) the predicted drinking speed was 523 ± 8.30 mL/min, and at the highest THI (79.3) 491 ± 8.12 mL/min.

The analyses of feeding behavior data integrated with demographic and weather records across 6 years showed that environmental and biological factors explain approximately half of the variation in daily milk consumption and drinking speed in preweaning female Holstein calves. Regarding weather, higher THI did not show a reduction in milk consumption but THI did reduce drinking speed. Others have reported that elevated THI did not reduce milk consumption when barns were ventilated (Dado-Senn et al., 2022), even though heat stress increased respiration rates (Dado-Senn et al., 2020; Ferraro et al., 2021). Finally, weight is associated with muscular strength (NRC, 1971a) and energy for maintenance (NASEM, 2021). In this study, animals born with the lightest weights drank milk at a slower speed and consumed less milk than the heavier ones.

Calves require additional energy for thermoregulation when the temperature is below 15°C, and they require to dissipate heat when the temperature is above 26°C (NASEM, 2021). Therefore, milk consumption was expected to be inversely related to THI (Roland et al., 2016). Although small, the current analysis showed a positive association of THI on milk consumption. This observation can result from the controlled conditions at the barn, the milk allowance, and the young age of the calves. There was no increase in milk consumption, possibly because barn curtains were closed when cold and calves were already consuming enough milk to cover the additional energy required to maintain their body temperature.

Calves were housed in ventilated barns, which could have mitigated the effect of heat stress on milk consumption. Similarly, a previous study found that those calves that were provided cooling fans under elevated THI conditions did not reduce their milk consumption (Dado-Senn et al., 2022). The same study reported that milk consumption differences between heat-stressed and cooled calves were greater as they aged, which were not statistically significant until the fifth week (Dado-Senn et al., 2022). Nonetheless, this study only observed the animals before they reached 5 wk of age, when THI may have a milder effect on milk consumption due to a larger surface area to body weight ratio. Moreover, given that calves prefer milk (Cantor et al., 2022a), they may have consumed less concentrate when heat stressed. The respiration rate of calves responds to environmental temperature, and is faster when they experience heat stress (Dado-Senn et al., 2020) and slower under cold conditions (Roland et al., 2016). The model showed that THI was inversely related to drinking speed. Under heat stress, elevated respiration rates could have impaired the sucking pattern coordination, resulting in slower drinking speed (Lau and Schanler, 1996). For a more accurate understanding of the influence of THI in feeding behavior of calves there is a need to collect within-barn climatic variables in future studies.

Calves with low birth weights drank milk at the slowest speed and consumed less milk than heavier calves. It was expected that heavier animals would consume more milk because they require more energy for maintenance (NASEM, 2021). The sucking efficiency of human infants depends on muscular strength and is correlated with body weight and maturity (Lau and Schanler, 1996; Cunha et al., 2009). The current analysis showed that the group with birth weights in the lightest quartile had a significantly slower drinking speed than the other groups. Milk consumption (Jensen, 2004) and drinking speed (O'Driscoll et al., 2006) could have been influenced by competition at the feeder, where subordinate calves are displaced by dominant calves when a resource is limited. However, pen population was not available from January 2015 to February 2019. Future studies with AMF should consider analyzing the effect of calves per pen on daily feeding behavior.

The results showed that calves born from multiparous cows consumed more milk than those born from primiparous cows, independent from birth weight. Shivley et al. (2018) reported that calves born from higher parity dams had higher average daily gains, which can be a consequence of higher milk consumption. In the future including more dam factors such as calving ease and health status could potentially explain the differences in feeding behavior by parity in this analysis.

Furthermore, temperament, physical traits, social and physical environment, equipment, and milk feeding plan contribute to the variation in feeding behaviors (Nielsen, 1999; Neave et al., 2018). The genetic background of individual calves is also expected to influence these traits. Therefore, the same threshold cannot be applied for all animals across contexts. Integrating activity data and considering deviations from individual baselines into machine learning models could assist the detection of sick animals (Cantor and Costa, 2022; Conboy et al., 2022) and in the derivation of novel traits to be included in dairy cattle breeding programs.

The main limitation of this retrospective study was data availability and verifiability. The variables considered in the models were those that the farm routinely recorded in the herd management software. The farm does not record temperature and humidity inside the barns calving difficulty or enteric disease incidence unless they require antibiotic treatment. The lack of these data led to the omission of confounds that can introduce bias. Another source of bias could be the filters applied to select a sample of calves that minimized the amount of missing data. An additional limitation for the reported results is that records on concentrate intake were not available and milk composition was not routinely monitored. The models in this study did not include complex interactions for simplicity in interpreting the results. Finally, this study only looks at one location. The inclusion of different environmental conditions, management practices, milk feeding plans, and equipment would lead to more robust observations.

Looking at all factors together in the same model and with a large sample size allowed for the identification of differences in feeding behavior potentially unobservable in a farm setting. Future controlled experiments should confirm and describe associations between dam parity, birth weight, and feeding behavior. However, validation of the variables recorded by the AMF, their precision, and sources of variation are still needed to improve the quality of feeding behavior data. Next would be associating external and animal factors with variation in feeding behaviors in the face of stressors or sickness to identify resilient animals. Given the results of this study, we suggest considering data about the dam, birth conditions, daily weather, and milk feeding in the performance assessment of dairy calves in an AMF system.
